# Targeted ultra-deep sequencing reveals recurrent and mutually exclusive mutations of cancer genes in blastic plasmacytoid dendritic cell neoplasm

**DOI:** 10.18632/oncotarget.2223

**Published:** 2014-07-16

**Authors:** Albrecht Stenzinger, Volker Endris, Nicole Pfarr, Mindaugas Andrulis, Korinna Jöhrens, Frederick Klauschen, Udo Siebolts, Thomas Wolf, Philipp-Sebastian Koch, Miriam Schulz, Wolfgang Hartschuh, Sergij Goerdt, Jochen K. Lennerz, Claudia Wickenhauser, Wolfram Klapper, Ioannis Anagnostopoulos, Wilko Weichert

**Affiliations:** ^1^ Institute of Pathology, University Hospital Heidelberg, Germany; ^2^ Institute of Pathology, Charité University Hospital, Berlin, Germany; ^3^ Institute of Pathology, University Hospital Halle and Institute of Pathology, University Hospital Leipzig, Germany; ^4^ Department of Dermatology, Venereology and Allergology, University Medical Center and Medical Faculty Mannheim, University of Heidelberg, Mannheim, Germany; ^5^ German Red Cross Blood Service and Institute for Transfusion Medicine and Immunohematology, Goethe University Medical School, Frankfurt, Germany; ^6^ Department of Dermatology, University Hospital Heidelberg, Germany; ^7^ Institute of Pathology, University of Ulm, Ulm, Germany; ^8^ Department of Pathology, Hematopathology Section and Lymph Node Registry, Christian-Albrechts-University of Kiel, Germany; ^9^ National Center for Tumor Diseases, Heidelberg, Germany; ^10^ Present address: Harvard Medical School, Massachusetts General Hospital, Department of Pathology, Boston, MA, USA

**Keywords:** blastic plasmacytoid dendritic cell neoplasm, BPDCN, recurrent mutations, mutually exclusive mutations, next generation sequencing, ATM, KRAS, NRAS, CDKN2A

## Abstract

Blastic plasmacytoid dendritic cell neoplasm (BPDCN) is a rare haematopoietic malignancy characterized by dismal prognosis and overall poor therapeutic response. Since the biology of BPDCN is barely understood, our study aims to shed light on the genetic make-up of these highly malignant tumors. Using targeted high-coverage massive parallel sequencing, we investigated 50 common cancer genes in 33 BPDCN samples. We detected point mutations in NRAS (27.3% of cases), ATM (21.2%), MET, KRAS, IDH2, KIT (9.1% each), APC and RB1 (6.1% each), as well as in VHL, BRAF, MLH1, TP53 and RET (3% each). Moreover, NRAS, KRAS and ATM mutations were found to be mutually exclusive and we observed recurrent mutations in NRAS, IDH2, APC and ATM. CDKN2A deletions were detected in 27.3% of the cases followed by deletions of RB1 (9.1%), PTEN and TP53 (3% each). The mutual exclusive distribution of some mutations may point to different subgroups of BPDCN whose biological significance remains to be explored.

## INTRODUCTION

Blastic plasmacytoid dendritic cell neoplasm (BPDCN) is an orphan disease with a very aggressive clinical course resulting in median survival times of 12-14 months [1,2]. The male-to-female sex ratio is approximately 2.0-3.5:1 and the mean age at diagnosis is 65-70 years. There is no standardized therapeutic approach [3] and despite initial response to chemotherapy, patients show a very high relapse rate, which points to an overall low efficacy of current therapeutic regimens [4-8].

Initially named blastic NK/T cell lymphoma or agranular CD4+/CD56+ hematodermic neoplasm/tumour [6, 9, 10, 11], BPDCN is –according to the current WHO classification- classified as an acute myeloid leukemia-related precursor neoplasm [12] that has been shown to derive from precursors of plasmacytoid dendritic cells [13, 14,15, 16]. The genetic basis of this malignancy, however, whose comprehension may yield new and more efficient therapeutic strategies, remains largely enigmatic [17]. As with other rare diseases, the low prevalence of BPDCN and its only quite recent recognition as a distinct tumour entity are major obstacles for comprehensive and systematic clinical and biological research efforts. Consequently, until now only few studies with limited numbers of cases have focused on the genetic foundations of BPDCN, mainly investigating copy number variations of tumor suppressor genes including TP53 and RB1 [18, 19, 20, 21]. Two more recent studies [18, 22] also addressed somatic point mutations in the genome of BPDCN. To complement previous efforts and to gain more insight into the genetic basis of BPDCN, we performed targeted ultra-deep semiconductor-based massive parallel sequencing of 50 cancer-related genes [23] in 33 formalin-fixed, paraffin embedded primary BPDCN samples and additionally assayed CDKN2A copy numbers.

## RESULTS

Thirty-three cases of BPDCN were analyzed for somatic mutations of 50 common cancer genes by ultra-deep targeted semiconductor-based massive parallel sequencing. A detailed overview of detected SNPs including allele frequencies is given in supplementary table 1. Patient demographics are described in table 1. Twenty-seven patients (81.8%) were male and the median age was 72 years. Except for one case with manifestation in the nasopharynx, all biopsy specimens were obtained from cutaneous infiltrates.

**Table 1:** Basic clinical characteristics of BPDCN cases

ID No.	Age [y]	Sex	Origin of tissue
01	77	f	skin
02	85	m	skin
03	81	m	skin
04	83	m	skin
05	60	m	skin
06	69	m	skin
07	73	m	skin
08	79	m	skin
09	67	m	skin
10	81	m	skin
11	76	m	skin
12	69	m	skin
13	30	m	skin
14	69	m	skin
15	83	m	skin
16	71	f	skin
17	82	m	skin
18	76	m	skin
19	54	f	skin
20	64	f	skin
21	83	m	skin
22	89	m	skin
23	68	f	nasopharynx
24	87	m	skin
25	60	m	skin
26	76	m	skin
27	72	m	skin
28	65	m	skin
29	60	m	skin
30	80	m	skin
31	52	f	skin
32	61	m	skin
33	72	m	skin

f: female, m: male; Age: age at diagnosis, y: years

**Figure 1:**
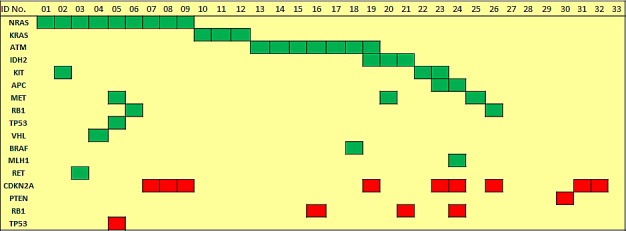
Distribution of recurrent and mutually exclusive point mutations (green color) in known cancer genes as well as of deletions (red color) in CDKN2A, PTEN, TP53 and RB1 across BPDCN cases.

**Figure 2:**
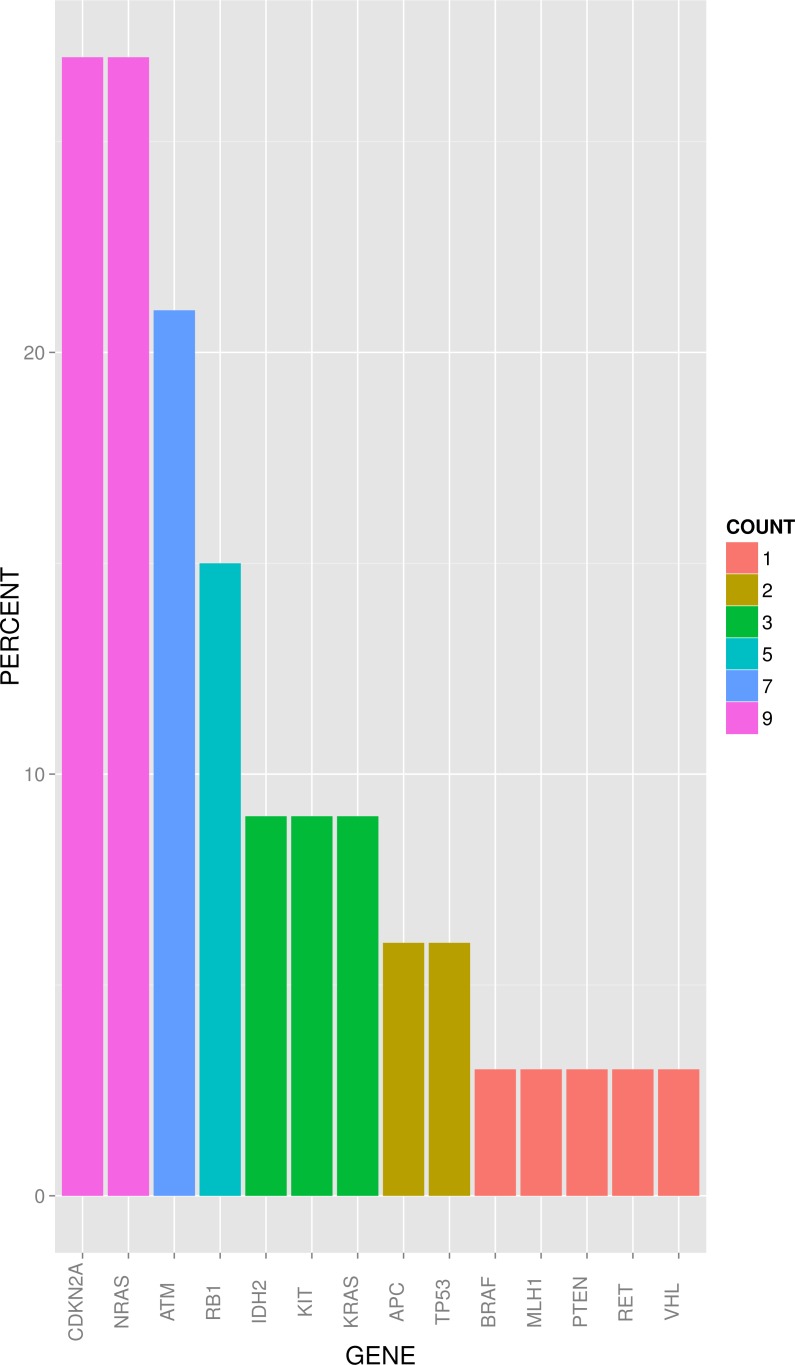
Frequency of genetic alterations (SNP and CNV) of known cancer genes in BPDCN (bar plot) in percent (descending order). COUNT indicates absolute numbers of cases.

Sequence analysis performed with an average read depth of 1.552-fold identified point mutations in 14 genes and deletions in 4 genes. As depicted in figure 1, 4 cases (12.1%) showed no detectable genetic alteration in the 50 genes investigated. For the other 29 cases (87.8%), the most frequent non-synonymous point mutations were found in NRAS (9/33 cases; 27.3%) and ATM (7/33; 21.2%). Point mutations at lower frequencies were detected in MET, KRAS, IDH2 and KIT (3/33; 9.1% each), APC and RB1 (2/33; 6.1% each), and VHL, BRAF, MLH1, TP53 and RET (1/33; 3% each). Deletions were most prevalent in CDKN2A (9/33; 27.3%) followed by RB1 (3/33; 9.1%), PTEN and TP53 (1/33; 3%). For CDKN2A deletions we performed a copy number assay to exemplarily validate our sequencing data by an independent method and corroborated all CDKN2A deletions detected by sequencing analysis. Figure 2 summarizes the frequency of genetic alterations (SNP and CNV combined) per gene.

Mutations in either RAS genes or ATM were present in 57.6% of cases and in a mutually exclusive distribution (figure 1). Notably, 16 (48.5%) cases displayed somatic alterations in two or more genes.

**Figure 3:**
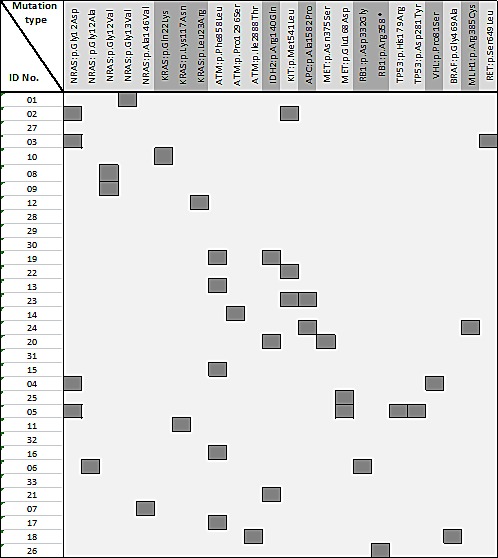
Distribution of non-synonymous somatic mutation types in known cancer genes across BPDCN samples. Amino acid substitutions as indicated.

Figure 3 depicts the type of point mutation and resulting amino acid substitution for all BPDCN specimens showing recurrent mutations in NRAS, IDH2, APC and ATM. Except for a nonsense mutation in RB1, mutations in the other genes were of missense type.

Interestingly, as depicted in figures 1 and 3, one case showed concomitant deletion of one TP53 allele and a double mutation in the remaining TP53 allele and we also found two exceedingly rare mutations in KRAS (p.Gln22Lys and p.Leu23Arg), which have been documented in Cosmic but not reported in the literature yet.

As expected, all mutations in genes coding for the MAPK cascade i.e. KRAS, NRAS genes and BRAF as well as point mutations in the tumor suppressor genes APC, VHL, RB1 and TP53 were classified as deleterious/damaging by four independent in silico tools (table 2). While mutations in MET and KIT as two potential drug targets were predicted as neutral, we found deleterious missense mutations in ATM and RET, both encoding proteins of the DNA damage response.

**Table 2:** In silico characterization of gene mutations including COSMIC annotations

Gene/Mutation	MutationTaster	PolyPhen	SIFT	PROVEAN	COSMIC
NRAS: p.Gly12Asp	Disease causing	possibly damaging	Damaging	Deleterious	COSM564 (n=450; 324 samples in haematopoietic and lymphoid tissue: i. a. leukemia, myelodysplastic syndrome), COSM46495 (n=4, leukemia)
NRAS: p.Gly12Ala	Disease causing	possibly damaging	Damaging	Deleterious	COSM565 (n=52; 36 samples in haematopoietic and lymphoid tissue: i. a. leukemia, BPDCN)
NRAS: p.Gly12Val	Disease causing	possibly damaging	Damaging	Deleterious	COSM566 (n=72; 55 samples in haematopoietic and lymphoid tissue: i. a. leukemia, myeloproliferative disease)
NRAS: p.Gly13Val	Disease causing	probably damaging	Damaging	Deleterious	COSM574 (n=61; 40 samples in haematopoietic and lymphoid tissue: i.a. leukemia, myelodysplastic syndromes, myeloproliferative disease, lymphoma) COSM46502 (n=1, leukemia)
NRAS: p.Ala146Val	Disease causing	probably damaging	Damaging	Deleterious	No COSMIC entry
KRAS: p.Gln22Lys	Disease causing	probably damaging	Damaging	Deleterious	COSM543 (n=9; 1 sample in haematopoietic and lymphoid tissue: leukemia)
KRAS: p.Leu23Arg	Disease causing	probably damaging	Damaging	Deleterious	COSM303853 (n=1; 1 sample in haematopoietic and lymphoid tissue: leukemia)
KRAS: p.Lys117Asn	Disease causing	probably damaging	Damaging	Deleterious	COSM28519 (n=6; 2 samples in haematopoietic and lymphoid tissue: leukemia)
ATM: p.Phe858Leu	Polymorphism	possibly damaging	Tolerated	Deleterious	COSM21826 (3 samples in haematopoietic and lymphoid tissue: Lymphoma)
ATM: p.Pro1296Ser	Polymorphism	probably damaging	Damaging	Deleterious	no COSMIC entry
ATM: p.Ile2888Thr	Disease causing	probably damaging	Damaging	Deleterious	COSM21679 (2 samples in haematopoietic and lymphoid tissue: leukemia, lymphoma)
IDH2: p.Arg140Gln	Disease causing	probably damaging	Damaging	Deleterious	COSM41590 (n=379; 378 samples in haematopoietic and lymphoid tissue: i. a. BPDCN, myeloproliferative disease, myelodysplastic syndrome, Leukemia)
KIT: p.Met541Leu	Polymorphism	benign	Tolerated	Neutral	COSM28026 (n=31; 16 samples in haematopoietic and lymphoid tissue: pediatric Mastocytosis, myeloid malignancies)
APC: p.Ala1582Pro	Disease causing	probably damaging	Damaging	Deleterious	no COSMIC entry
MET: p.Glu168Asp	Polymorphism	possibly damaging	Tolerated	Neutral	COSM706 (n=2), COSM29811 (n=4; 1 sample in haematopoietic and lymphoid tissue: Langerhans cell histiocytosis)
MET: p.Asn375Ser	Disease causing	benign	Tolerated	Neutral	COSM710 (n=3), COSM28925 (n=18)
RB1: p.Asp332Gly	Disease causing	probably damaging	Damaging	Deleterious	no COSMIC entry
RB1: p.Arg358*	Disease causing	Nonsense	Nonsense	Nonsense	COSM879 (n=6)
TP53: p.His179Arg	Disease causing	probably damaging	Damaging	Deleterious	COSM10889 (n=114), COSM87198 (n=5)
TP53: p.Asp281Tyr	Disease causing	probably damaging	Damaging	Deleterious	COSM11516 (n=10; 1 sample in haematopoietic and lymphoid tissue: lymphoma)
VHL: p.Pro81Ser	Disease causing	possibly damaging	Damaging	Deleterious	COSM17721 (n=16) COSM144163 (n=1)
BRAF: p.Gly469Ala	Disease causing	probably damaging	Damaging	Deleterious	COSM460 (n=31; 6 samples in haematopoietic and lymphoid tissue: leukemia, lymphoma), COSM29608 (n=21)
MLH1:p.Arg385Cys	Disease causing	probably damaging	Damaging	Deleterious	COSM1422593 (n=1)
RET:p.Ser649Leu	Disease causing	probably damaging	Damaging	Neutral	no COSMIC entry

## DISCUSSION

Here, we performed targeted high-coverage massive parallel sequencing of 50 common cancer genes in 33 cases of BPDCN. We found recurrent mutations in 4 cancer genes and delineated that NRAS, KRAS and ATM mutations are distributed in a mutually exclusive fashion.

BPDCN is a rare and aggressive hematologic malignancy, whose pathobiology is still largely obscure. Over the last 5 years studies on the genetic foundations of BPDCN revealed a peculiar genotype with a complex karyotype and recurrent deletions in several tumor suppressor genes, such as RB1, TP53, CDKN2A and CDKN2B [19, 20,21, 28]. While the data on copy number variations seem to be rather specific for BPDCN among hematologic neoplasms, recent work [18, 29] demonstrated point mutations in TET2 und TP53 in the genome of BPDCN thereby genetically relating BPDCN to a broad range of myeloid malignancies, where recurrent mutations particularly in the tumour suppressor TET2 but also in TP53 are highly prevalent [30, 31]. In accord with these reports, we detected deletions, which were most prevalent for CDKN2A and less frequent for RB1, TP53 and PTEN. Moreover we found point mutations in TP53, IDH2, KRAS and NRAS further underpinning a genetic relation between BPDCN and myeloid malignancies, including acute myeloid leukemia and myelodysplastic syndrome. Thereby, our findings extend the observations of prior studies and support the genetic rationale for the current WHO classification of BPDCN as a distinct precursor neoplasm that relates to acute myeloid leukemia. In this context it is notable that we did not find mutations in other genes that have been described in myeloid malignancies, such as FLT3, NPM1, IDH1, JAK2, and EZH2. A very recent study by Menezes *et al* [22], who used a combined approach of whole exome sequencing-based identification of major genetic aberrations and targeted resequencing of 25 BPDCN, found similar frequencies of mutations in NRAS and corroborated TET2 mutations albeit at a lower frequency as reported previously [18, 29]. Additionally, they identified mutations in genes not included in our gene panel, namely in ASXL1 and in the transcription factors IKZF1-3 and ZEB2. Taken together, these findings strongly support the view that BPDCN is a distinct entity within the spectrum of myeloid malignancies.

Interestingly, we discovered missense mutations in DNA damage response genes, i.e. MLH1 and ATM in a substantial number of cases. Of three ATM variants, we have detected in our cohort, two are already reported in the Cosmic database and all three are classified as damaging by the majority of the in silico prediction models used herein. ATM aberrations have been mainly implicated in the development of lymphomas, including T-cell prolymphocytic leukemia [32], mantle cell lymphoma [33], diffuse large B-cell lymphoma [34] and B-cell chronic lymphocytic leukemia [35]. Although we do not know yet what these ATM mutations actually mean for BPDCN biology, In the light of the above mentioned studies, our genetic data may point to a shared trait with lymphoid malignancies. In line with these findings, BPDCN express also molecules that can be found in malignant lymphomas, such as CD4 and CD123 and it has been demonstrated in mice that normal plasmacytoid dendritic cells retain remarkable differentiation plasticity and can derive from both myeloid and lymphoid progenitors [36]. Also, a recent study conducted by Sapienza *et al* [37] found BPDCN to display an ambiguous expression profile that indeed related to both acute lymphoid and acute myeloid leukemia. Moreover, although these data do not stem from randomized clinical trials and therefore have to be interpreted carefully, several clinical reports have demonstrated a considerable efficacy of acute lymphoid leukemia-like protocols in BPDCN treatment [38, 39].

While it is well known that RAS signaling plays a role in other hematologic malignancies, our study shows for the first time that BPDCN specimens have mutually exclusive mutations in NRAS and KRAS and recurrent mutations in NRAS. Our study suggests that the prevalence of RAS mutations in approximately 35% of BPDCN even exceeds the ones reported for other hematological neoplasms known to harbor RAS-mutations, namely juvenile myelomonocytic leukemia (JMML), chronic myelomonocytic leukemia (CMML), acute myeloid leukemia (AML), myelodysplastic syndrome (MDS), acute lymphoblastic leukemia (ALL) and multiple myeloma (MM) [40]. As already reported for these neoplasms, BPDCN also shows a clear predominance of NRAS mutations over KRAS mutations. Although tremendous research efforts are being made, powerful and clinically effective inhibition of mutated Ras is still at a distant prospect. However, given the high prevalence of mutated RAS genes, targeting RAS or downstream effectors of the MAPK-pathway such as MEK and ERK [41, 42] would be a valuable treatment option for BPDCN.

Since mutations in NRAS and KRAS as well as in ATM were found to be recurrent and mutually exclusive, it is tempting to speculate whether these data point to genetically distinct subgroups within the BPDCN phenotype.

At this point the lack of reliable BPDCN models precludes functional analysis or the study of biological implications of our genetic data. Furthermore, the overall rarity of BPDCN precludes analysis of clinical course or outcome data. Nonetheless, our sample size of 33 primary tumour samples at least equals or exceeds the number of specimens in prior studies [18, 22]. In conjunction with these studies, here, we extend the genetic framework of BPDCN, which is an important step in understanding the pathobiology of BPDCN and may also aid in finding novel therapeutic options for this orphan disease.

## Material and Methods

### Ethics statement

This study was conducted as an anonymized case- and specimen review via the tissue bank of the National Center for Tumor Diseases (NCT, Heidelberg, Germany). All experiments were performed in accordance with the Declaration of Helsinki.

### Samples

Formalin-fixed, paraffin embedded tissue from 33 cases of BPDCN were obtained from the Institute of Pathology and Department of Dermatology, University Hospital Heidelberg, the Institute of Pathology, Charité University Hospital, Berlin, the Institute of Pathology at the University of Leipzig, the Department of Pathology, Christian-Albrechts-University of Kiel, and the Department of Dermatology, Venereology and Allergology, University Medical Center and Medical Faculty Mannheim, University of Heidelberg. All cases were diagnosed by experienced haematopathologists (AM, IA, KJ, WK, CW) according to the criteria of the World Health Organization classification of heamatopoietic and lymphoid tissue [12]. Patient data were anonymized. Due to the fact that the majority of cases had been sent for consultation no follow-up data were available.

### DNA extraction

Extraction of genomic DNA was performed by proteinase K digestion and fully automated purification using the QIASymphonySP (Qiagen, Hilden, Germany). The total DNA content was measured using the QuBit HS DNA Assay (Life Technologies, Darmstadt, Germany), followed by measuring the amount of amplifiable DNA using quantitative PCR [23].

### Library preparation and semiconductor sequencing

For this study the multiplex PCR based Ion Torrent AmpliSeq cancer Hotspot panel version 2 (Life Technologies, Darmstadt, Germany) was used, covering approx. 2,800 COSMIC annotated mutations in the following 50 key cancer genes: ABL1, AKT1, ALK, APC, ATM, BRAF, CDH1, CDKN2A, CSF1R, CTNNB1, EGFR, ERBB2, ERBB4, EZH2, FBXW7, FGFR1, FGFR2, FGFR3, FLT3, GNA11, GNAS, GNAQ, HNF1A, HRAS, IDH1, IDH2, JAK2, JAK3, KDR, KIT, KRAS, MET, MLH1, MPL, NOTCH1, NPM1, NRAS, PDGFRA, PIK3CA, PTEN, PTPN11, RB1, RET, SMAD4, SMARCB1, SMO, SRC, STK11, TP53, and VHL. Amplicon library preparation was performed using approximately 10 ng of DNA as advised by the manufacturer. Briefly, the DNA was mixed with the primer pool, containing all primers for generating the 207 amplicons, and the AmpliSeq HiFi Master Mix and transferred to a PCR cycler (Biometra, Göttingen, Germany). PCR cycling conditions were as follows: Initial denaturation: 99°C for 2 min, cycling: 21 cycles of 99°C, 15 sec and 60°C, 4 min. After the end of the PCR reaction, primer end sequences were partially digested using FuPa reagent as instructed, followed by the ligation of barcoded sequencing adapters (Ion Xpress Barcode Adapters, Life Technologies, Darmstadt, Germany). The final library was purified using AMPure XP magnetic beads (Beckman Coulter, Krefeld, Germany) and quantified using qPCR (Ion Library Quantitation Kit, Life Technologies, Darmstadt, Germany) on a StepOne Plus Instrument (Life Technologies, Darmstadt, Germany). The individual libraries were diluted to a final concentration of 100pM and eight to ten libraries were pooled and processed to library amplification on Ion Spheres using the Ion OneTouch 2 instrumentation with the 200 bp chemistry. Unenriched libraries were quality-controlled using Ion Sphere quality control measurement on a QuBit instrument. After library enrichment (Ion OneTouch ES), the library was processed for sequencing using the Ion Torrent 200 bp sequencing chemistry and the barcoded eight to ten libraries were loaded onto a single 318 chip.

### Data analysis

Raw data analysis was performed using Ion Torrent Software Suite (Version 3.6 and 4.0, respectively). The reads were aligned to the human reference sequence build 38 (hg19) using the TMAP aligner implemented in the Torrent Suite software. Detection of single base pair variants and insertion-deletion polymorphisms (InDels) compared to the human reference sequence was performed using either Ion Torrent Variant Caller (3.6 and 4.0). Detection thresholds for SNPs and InDels were set at an allele frequency of 5%. Variants were annotated and filtered against the dbSNP and COSMIC databases and screened for possible splice site effect using the CLC genomics Suite 6 (CLCbio, Aarhus, Denmark). Copy number variation was determined using the coverage analysis plug-in of the Torrent Suite software.

### Copy number alterations

All tissue samples were analyzed for CDKN2A copy number using a Taqman® copy number assay (Life Technologies, Carlsbad, CA) to measure copy number variation at the CDKN2A locus. The assay is a duplex polymerase chain reaction (PCR) for the CDKN2A gene and the reference gene, RNaseP (normalizer), using 10 ng DNA in quadruplicate PCR according to the manufacturer's protocol and run on the StepOnePlus real time PCR instrument (Life Technologies). The results were calculated as a ratio relative to a 2-copy control using the Copy Caller software (Life Technologies). Loss of CDKN2A was defined as ≤1.5 copies, no loss was >1.5 copies.

### In silico analysis of mutations

The biological impact of the mutations on the structure and function of the respective protein product was predicted in silico by the use of four different software tools: Provean (http://provean.jcvi.org/index.php) [24], Sift (http://sift.jcvi.org/) [25], MutationTaster (http://www.mutationtaster.org/) [26] and PolyPhen (http://genetics.bwh.harvard.edu/pph/data/) [27]. Additionally we used the COSMIC (catalogue of somatic mutations in cancer) database to check our sequencing data for somatic mutations that have already been reported elsewhere.

### Author contributions

V.E., N.P. and A.S. performed analysis of the sequence data. T.W., J.K.L. and M.S. contributed to data analysis. A.S. and W.W. coordinated sample acquisition and processing. P.K., M.A., K.J., U.S., W.H., S.G., C.W. and W.K. provided samples and clinical data. A.S., V.E., I.A. and W.W. wrote the manuscript, with contributions from M.A., F. K. and J.K.L. A.S., V.E., W.W. and I.A. directed the research.

## SUPPLEMENTARY TABLES


